# Qualitative evaluation of a pilot educational intervention to increase primary care HIV-testing

**DOI:** 10.1186/s12875-019-0962-3

**Published:** 2019-05-31

**Authors:** Joanna M. Kesten, Charlotte F. Davies, Mark Gompels, Megan Crofts, Annette Billing, Margaret T. May, Jeremy Horwood

**Affiliations:** 1 National Institute for Health Research (NIHR) Health Protection Research Unit in Evaluation of Interventions, Population Health Sciences, Bristol Medical School, University of Bristol, Oakfield House, Oakfield Grove, Bristol, BS8 2BN UK; 2 NIHR Collaborations for Leadership in Applied Health Research and Care West (CLAHRC West), University Hospitals Bristol, NHS Foundation Trust, 9th Floor, Whitefriars, Lewins Mead, Bristol, BS1 2NT UK; 3 Population Health Sciences, Bristol Medical School, University of Bristol, Canynge Hall, 39 Whatley Road, Bristol, BS8 2PS UK; 4 Department of Immunology, Southmead Hospital, North Bristol NHS Trust, Westbury-on-Trym, Bristol, BS10 5NB UK; 5 Genitourinary medicine, Unity Sexual Health, Bristol Sexual Health Services, Tower Hill, Bristol, BS2 0JD UK; 6 Bristol, North Somerset and South Gloucestershire CCG, South Plaza, Marlborough Street, Bristol, BS1 3NX UK

**Keywords:** HIV testing, Primary care, Educational intervention, Qualitative research

## Abstract

**Background:**

UK guidelines recommend a ‘routine offer of HIV testing’ in primary care where HIV diagnosed prevalence exceeds 2 in 1000. However, current primary care HIV testing rates are low. Efforts to increase primary care HIV testing are needed. To examine how an educational intervention to increase HIV testing in general practice was experienced by healthcare professionals (HCPs) and to understand the perceived impacts on HIV testing.

**Method:**

Qualitative interviews with general practitioners (GPs) and nurses 3-months after receiving an educational intervention developed from an adapted version of the Medical Foundation for HIV and Sexual Health (MEDFASH) HIV Testing In Practice (TIPs) online educational tool which included training on HIV associated clinical indicator conditions, why, who, and how to test. The intervention was delivered in 19 high-HIV prevalence general practices in Bristol. 27 semi-structured interviews were conducted across 13 practices with 16 GPs, 10 nurses and the sexual health clinician who delivered the intervention. Transcripts were analysed thematically informed by Normalisation Process Theory.

**Results:**

HCPs welcomed the opportunity to update their HIV knowledge through a tailored, interactive session. Post-training, HCPs reported increased awareness of HIV indicator conditions, confidence to offer HIV tests and consideration of HIV tests. Continued testing barriers include perceived lack of opportunity.

**Conclusions:**

This qualitative study found that HIV education is perceived as valuable in relation to perceived awareness, confidence, and consideration of HIV testing. However, repetition and support from other strategies are needed to encourage HCPs to offer HIV tests. Future interventions should consider using behaviour change theory to develop a complex intervention that addresses not only HCP capability to offer an HIV test, but also issues of opportunity and motivation.

## Background

The UK Royal College of General Practitioners named sexual health a priority area for improvement [1]. Efforts to increase testing for human immunodeficiency virus (HIV) within primary care [2–4] are specifically needed [5, 6] to address the proportion of people living with undiagnosed [7] and late diagnosed HIV [8, 9]. Patients often present to primary care with a related condition several times before being diagnosed with HIV (referred to as HIV indicator conditions (HIV ICs) [2, 3, 10, 11]. Delayed and late diagnosis is associated with negative outcomes for patients (greater chance of being hospitalised, reduced life expectancy [12–14]) and greater treatment and hospital care costs [15]. Contrastingly, anti-retroviral therapy increases life expectancy and effectively reduces infection transmission risk among treated individuals [16].

Healthcare professionals (HCPs) offering HIV tests could increase testing rates [17] and reduce undiagnosed and late diagnosed infection in the UK [8, 18, 19]. This is in line with UK guidance which recommends routine general practice HIV testing in areas with an HIV diagnosed population prevalence of > 2 per 1000 [20–23]. The guidance recommends offering a test to all new practice patients [23] and to patients presenting with HIV ICs [19]. However, barriers to HIV testing experienced by HCPs include gaps in knowledge and training needs [17, 24, 25], anxiety or a lack of confidence to offer a test, concern about negative patient response [25, 26] and, privacy and confidentiality issues [25, 26]. These barriers and the opportunity to diagnose HIV through improved recognition of HIV ICs indicate a role for educational interventions to encourage increased testing [11]. Such interventions have shown encouraging results in high-HIV prevalence UK cities [25, 27–29].

We aimed to understand how an educational intervention to increase HIV testing in primary care was experienced and to explore the perceived effect on HIV testing. A quantitative evaluation of the intervention is reported separately [30].

## Methods

### Research setting

GP practices in Bristol, South Gloucestershire and North Somerset with high practice population HIV prevalence (> 2 per 1000).

### Study design

Qualitative study nested within a stepped-wedge pilot randomised controlled trial of an educational intervention to increase HIV testing. The intervention consisted of a one-hour interactive training workshop delivered in 19 general practices in Bristol to 169 HCPs (93 GPs, 53 nurses and 23 `others’), by a genitourinary medicine (GUM) specialist registrar. Intervention practices received the training between October 2015 and March 2016 and control practices from April to July 2016. The training was developed from an adapted version of the Medical Foundation for HIV and Sexual Health (MEDFASH) HIV Testing In Practice (TIPs) online educational tool (http://www.medfash.org.uk/welcome-to-hiv-tips). It consisted of a short quiz assessing current knowledge, an update on current British HIV Association (BHIVA) and the National Institute for Health and Care Excellence (NICE) HIV testing recommendations and HIV ICs, particularly focusing on common presentations in primary care (e.g. HIV ICs, seroconversion illness). The training also covered: the importance of HIV testing, who to test, how to discuss an HIV test with the patient, ways to overcome barriers to testing, how to manage negative and positive results and linkage to care. The training concluded with a discussion, supported by case study examples, about ways to increase practice level testing. The content was locally tailored, signposting to relevant statistics and services. Following the training, each practice received summary information and references to useful resources.

HCPs within each GP practice were invited by email in batches (with a written participant information sheet) approximately 3-months after receiving the intervention to participate in a semi-structured telephone or face-to-face interview. Interviews continued until theoretical saturation of key concepts had been reached [31]. Conducting interviews within several practices elucidated an understanding of contextual factors which supported or inhibited the effects of the intervention.

Interviews explored pre-training HIV testing knowledge and factors influencing testing, views of the training and, perceived impact on knowledge, confidence and testing practices. Practices were reimbursed £40 per interview.

With informed consent (written or audio for telephone interviews) interviews were audio recorded, transcribed verbatim and analysed thematically [32] using QSR NVivo10. The data were initially coded and a subset of transcripts independently analysed by JK and JH, to contribute to the refinement of codes. Codes were built into broader categories through comparison across transcripts and higher-level themes developed. The Normalisation Process Theory (NPT) constructs [33] were used to further develop themes. NPT proposes that implementation of interventions is dependent on the ability of participants to fulfil four criteria: 1. Coherence (HCPs views and understanding of the content and purpose of the intervention and their part within it); 2. Cognitive participation (HCPs’ engagement and views of the intervention and their role in implementation); 3. Collective action (HCPs’ actions to push the intervention forwards); 4. Reflexive monitoring (appraisal of the intervention) [33].

All HCPs who had not opted out of an interview invitation (*n* = 21) were eligible to participate. 104 HCPs across 18 out of 19 practices were invited to take part. In total, 26 HCPs (16 GPs, 10 nurses/healthcare assistant/advanced nurse practitioner, 5 males, 17 years of experience on average ranging from 1 to 36 years) from 13 GP practices agreed to participate in interviews lasting 30 min on average (Table 1) between March and September 2016. The proportion of GPs and nurses interviewed is reflective of the proportion of HCPs in these roles receiving the intervention. The intervention deliverer was also interviewed. 20 interviews were conducted by telephone and seven face-to-face. Findings are presented for each of the NPT constructs (Table 2) accompanied by anonymised illustrative quotes.

**Table 1:** Interview participant characteristics

General Practice Number	GP (n)	Nurses & Other^a^(n)	Gender of HCP interviewed Male/Female	Interviews Total (n)
1	1	1	1/1	2
2	0	1	0/1	1
3	0	1	0/1	1
4	2	2	0/4	4
5	0	2	1/1	2
6	2	0	1/1	2
7	1	0	0/1	1
8	0	1	0/1	1
9	6	1	2/5	7
10	0	1	0/1	1
11	1	0	0/1	1
12	2	0	0/2	2
13	1	0	0/1	1
Total				26

^a^Advanced nurse practitioner and Healthcare Assistant

**Table 2:** Overview of themes and subthemes for each Normalisation Process Theory (NPT) construct

NPT Construct	Theme	Subtheme
Coherence		
	Pre-training HIV testing	
		Testing situations (e.g. opportunistic, patient characteristics and behaviours, appointment type, consultation presentation with “obvious” indicator conditions)
		Perceived adequacy
		Frequency
		Nurse vs GP testing
		Patient acceptability
		Confidence to offer a test
	Pre-training HCP knowledge	
	Perceived need for training	
Cognitive participation		
	Experience of training	
		Content (e.g. appropriate to knowledge level)
		Delivery (e.g. location, length, interactive format)
	Impact of training	
		Intentions to change testing practice
		Awareness and knowledge (e.g. need to test early, indicator conditions, HIV prevalence in practice population)
		Confidence to test
Collective action		
	When to test	
		HIV testing normalised / viewed as routine
		Increased consideration of HIV testing
		Presence of indicator conditions and atypical, unexplained and persistent infections
		Patient response to offer of HIV test
		Nurses and Healthcare Assistants empowered to offer HIV tests
		Changes in number of HIV tests
	How testing is offered	
		Language used in consultation
		Pre-test counselling and consent
Reflexive monitoring		
	Barriers to HIV testing	
		Presence of relatives in consultation
		Language barriers
		Multiple problems to address in consultation
		Changing the subject to HIV / phrases to use
		Appointment time constraints
		Lack of opportunity to test
		Patient acceptability and agenda
	Improvements to training	
		Role play exercises and case examples
		More information on managing positive results
		Length of training
		Follow-up training and email reminders
		Primary care system changes (e.g. computer prompts, and universal screening)

## Results

### Coherence

Before the educational intervention, HIV testing was described as ad-hoc, opportunistic and informed by patient characteristics (e.g. high-risk groups such as men-who-have-sex-with-men, people who inject drugs and country of origin with high-HIV prevalence) and behaviours (e.g. unprotected sex), appointment type (e.g. sexual health screening and antenatal appointments) and consultation presentation with “obvious” indicator conditions (e.g. rash, chronic diarrhoea). Some HCPs felt that individual and practice level HIV testing was adequate while others reflected that more testing was needed. Several HCPs described offering HIV tests infrequently (i.e. 1–2 tests per year) while others tested routinely. Some nurses described only doing HIV tests requested by GPs.

*It was very ad hoc and a bit chaotic, and it would be really just if we thought of it.*

Practice 9, Female, Doctor, Interview 13

Pre-training, the acceptability of HIV testing among patients was perceived to vary and some HCPs were anxious about offering the test to some patients due to concern about appearing to judge and stigmatise.

If you’d have asked me that before the teaching I would’ve very much said the communication side, the getting across, and the worry that the patients would feel that I was judging them.

Practice 4, Female, Doctor, Interview 18

Most HCPs felt the training was necessary and relevant. However, two questioned the appropriateness of targeting their practices based on high-HIV prevalence because they perceived the incidence of new HIV cases as low.

*I think it’s [educational intervention] extremely necessary, (…) for our practice population it’s extremely relevant.*

Practice 7, Female, Doctor, Interview 4

*I think we actually have a very low [HIV] incidence because the vast amount [of patients] were diagnosed sort of 10 years ago (…) actually the prevalence may seem great but whether we were really a target audience is not quite as clear to me.*

Practice 9, Male, Doctor, Interview 20.

Some HCPs felt reasonably knowledgeable about HIV pre-training in part due to their practice population. While a few HCPs described lacking HIV knowledge (e.g. awareness of indicator conditions) and training.

*I would have thought we’re reasonably clued-up on this kind of thing because of the population we work in.*

Practice 4, Female, Doctor, Interview 14

### Cognitive participation

HCPs approved of the training’s interactive and informal style, which encouraged practice level discussion and supported increased awareness of HIV testing practice of peers. The training length and location was acceptable, however, some practices experienced difficulties organising a convenient time for staff due to variable working patterns. Well attended sessions were held within pre-existing staff meetings. The content was seen as appropriate to primary care and pre-existing knowledge. The local and specialist knowledge of the GUM specialist registrar delivering the intervention was valued. The intervention deliverer and a minority of participants described the training as increasing knowledge of local specialist services for patient referral and reciprocal relationships between primary and specialist sexual health care.

*I liked the way it was very informal, lots of opportunity to ask questions, that was really useful, but I loved the way she delivered these messages all the way through (…) she let people chat around the subjects and then she brought them to the conclusion.*

Practice 4, Female, Nurse, Interview 23

*It’s nice to know what service is available and there are specialists who would be able to help or give some advice.*

Practice 3, Female, Nurse, Interview 10

Most HCPs described intentions to change their practice in relation to HIV testing and adopt some of the messages from the training. The training helped HCP address the barrier of remembering when to offer an HIV test by increasing consideration of HIV. Most HCPs described feeling more aware of the need to test for HIV, the importance of testing early to improve prognosis and increased knowledge of both indicator conditions and HIV prevalence of their practice population. The training addressed HCPs concern about patients wanting detailed HIV information, by equipping them with more knowledge and verbal strategies to use. Most HCP felt more confident in their HIV knowledge, ability to talk to patients about HIV and to offer tests. In contrast, a minority did not feel more confident as this was not a pre-training issue.

*‘Think HIV’ so in a much wider set of conditions or symptoms, or situations, to actually be thinking about testing (…) I think it’s my thresholds and my internal alarm bells, probably have got a lower setting now than they did before.*

Practice 1, Male, Doctor, Interview 16

*I would probably now offer it more readily if needed rather than being panicky and scared about offering it.*

Practice 1, Female, Nurse, Interview 11

*I think it was just really ways (…) incorporate it into the consultation (…) if somebody was disclosing…you know if a man was disclosing to you that they’d you know had sex with another man or drug user etc., then how you can actually move that consultation on to say why we want to test and you know little scenarios of how you could ask you know, ask that and inform the patient – I thought that was really good.*

Practice 5, Female, Nurse, Interview 9

### Collective action

#### When to test

Following the training, HCPs were more likely to view HIV as a normal, routine test. Some HCPs described targeting testing toward patients with indicator conditions rather than focusing on those considered to be at increased risk. This reflected consideration of HIV testing for a *“wider spectrum of people”* and a *“lower threshold”* for offering the test, which in part was attributed to an increased awareness of indicator conditions. This led to it being viewed as a test to offer alongside other tests. Indeed, some GPs acknowledged that it can be difficult to ascertain whether patients are at high-risk of HIV which could prevent them from offering tests appropriately. However, some HCPs still felt the need to use patient characteristics and explicit or assumed risk factors to justify HIV testing and two GPs commented that it remained easier to offer an HIV test to high-risk patients.

*I think [the key take home message from the training is] don’t be afraid to ask the question or offer the test, because we always assume there are certain high-risk groups so we should just be focusing on them and in fact it’s out there, anybody could be affected, its perhaps where you least expect it.*

Practice 11, Female, Doctor, Interview 25

*I mean obviously for high-risk groups it’s different, I think about HIV a lot more, you know, if I see a homosexual man or an intravenous drug user, or a sex worker or something. Then I think about it, but in the general population I think about it far less.*

Practice 9, Female, Doctor, Interview 15

The training was experienced as increasing the likelihood of considering HIV as a diagnostic test for atypical, unexplained and persistent infections. In contrast, some HCPs queried the practicality of testing those with common indicator conditions (e.g. chronic tiredness).

*… Immune things or you know recurrent viral illnesses, recurrent bacterial infections, things that normally in my mind I’d jump straight to ‘oh we need to check they’re not diabetic’ are now also kind of jumping in to my mind ‘oh we need to check for HIV status as well’ which again is a change.*

Practice 4, Female, Doctor, Interview 18

The training reassured HCPs that patients are likely to find HIV testing acceptable and concerns about offending patients were reduced by highlighting that testing should be routine rather than targeted at high-risk groups. This helped address concerns about offering an HIV test as *“insinuating anything about their [patient] lifestyle”.* HCPs also felt more confident about allaying patient anxiety.

*They’ve taken it better than I originally maybe thought they had and maybe that’s why I have become more relaxed about it myself. … my own worry I think as I said at the beginning would be that they would feel that I was judging their behaviours and, but actually you know…maybe it’s the way that I’m now quite confident in putting it.*

Practice 4, Female, Doctor, Interview 18

*I think the training was quite good about encouraging us to normalise it [HIV testing] and you know opening it up to be a more frequent test that we do … and I think with that would follow that people expect it more and don’t consider it to be a judgmental or negative thing to be doing like it perhaps was in the past.*

Practice 12, Female, Doctor, Interview 8

The training led to nurses and healthcare assistants (HCAs) feeling more empowered and confident to offer HIV tests without referring patients to or asking a GP’s permission. Some HCPs perceived themselves and others to be offering more HIV tests, while others felt the training had limited impact on testing practice and they had not yet had the opportunity to test for HIV.

*Now I can make a decision of who I need to do it on and I don’t have to run to the GP and ask him all the time.*

Practice 1, Female, Nurse, Interview 11

*I’ve requested two patients have HIV tests since the training which is probably about the same as I’ve done in the year prior to that, so in other words I’ve increased my frequency of testing.*

Practice 9, Female, Doctor, Interview 21

*I would be keen to try and increase my screening but as, yet I haven’t sort of felt ‘oh this is a case where that’s relevant’.*

Practice 9, Male, Doctor, Interview 20

#### How testing is offered

Following the training, consultation communication about HIV testing was described as more *“forthright”*, *“matter of fact”*, *“informal”* and, *“less in-depth”.* Some HCPs felt that the time needed to offer an HIV test had reduced since the training because it highlighted that the test can be offered without lengthy counselling. For some, this represented a change in practice, which made offering a test easier and more efficient and enabled HIV tests to be offered in routine consultations for other conditions. In contrast, more experienced HCPs reflected that their own *“patter”* around HIV testing had not changed, while a minority argued that offering an HIV test could not be done quickly as informed consent had to be sought to ensure patients understand the test’s implications.

*In the past, one of the things you know if I did think of it, then it would be ‘how am I going to get this into the discussion? What other discussions are gonna be raised? We’re already 10-15 minutes into a 10-minute appointment … ’ whereas now I can say ‘well we need to check some bloods, as part of those blood tests I want to check your kidneys, your liver, and if it’s ok with you I’m also gonna add on an HIV test because that can also have quite a big impact on your physical health’ (…) it may well be negative but it’s better to check for it.’*

Practice 4, Female, Doctor, Interview 18

*I don’t think we’d have a leg to stand on if we did an HIV test on somebody without them knowing and it came back positive (…) so that I didn’t agree with her [intervention deliverer] (…) and because there’s stigma attached to HIV testing it’s particularly important that consent is documented.*

Practice 9, Female, Doctor, Interview 19

### Reflexive monitoring

Several barriers to HIV testing persisted after the training. Relatives and interpreters present in consultations or patients presenting with multiple issues made discussing HIV challenging. Similarly, although smear tests were viewed as a good HIV testing opportunity it was difficult to do both tests in the same consultation due to time constraints. Patients seeing multiple HCPs was another challenge for detecting relevant signs and symptoms reported previously. Some GPs felt it was unlikely that they would take bloods themselves in the consultation, instead some practices had a phlebotomy service and others asked patients to book in with a nurse. A couple of nurses described doing blood tests within the consultation rather than asking patients to make another appointment as they may not return. A few HCPs raised having continued concerns of introducing the topic of HIV when this was not the original reason for consulting. Managing patient expectations and the patient agenda was challenging in this situation. A minority of HCPs felt HIV testing was not part of their role either because they did not see ‘appropriate’ patient groups or believed GPs were responsible for these tests rather than nurses.

*You’ve got ten minutes, and the HIV testing is not top of your list. And so what I would normally do in that situation is tell them to come back and see the nurse or make another appointment for a sexual health screen, which I appreciate isn’t ideal, because you should seize the moment, but there’s only so many moments in a consultation and you’ve often run out by that point.*

Practice 4, Female, Doctor, Interview 14

Approximately half the interviewed HCPs had no suggestions for improving the training. A minority suggested role-play exercises or observing consultations in which HIV tests are offered and two suggested that more case examples would be valuable. A couple of HCPs wanted more guidance on dealing with positive test results and a few HCPs suggested a longer session.

Some HCPs commented that they struggled to remember the training content, beyond the key messages. *“Regular”*/ *“intermittent”* follow-up sessions were recommended by several HCPs to help them remember the content, consolidate learning and optimise the training’s impact.

HCPs suggested email reminders of the training content, computer system (e.g. pop-ups) and laboratory prompts as well as universal screening for all new registrants could support/increase HIV testing. Although computer prompts were seen as a good idea, one nurse queried how such a system would determine which patients to include a pop-up for or whether a universal pop-up system would be more appropriate. Too many or indiscriminate prompts may become ineffective. A minority of HCPs noted that the option to test for HIV was not easily visible on the computer system. Testing encouragement and feedback from the laboratory was expected to support HIV testing. One practice described how the laboratory had praised a HCP for testing for HIV and this had been shared within the practice as a good learning point. Automatically adding HIV tests for related blood tests were suggested. However, these measures were expected to have workload and financial implications. A more efficient system for giving results whereby receptionists phone patients with negative results was proposed. While, in some practices, all HIV test results were given by GPs which may limit the potential to increase HIV testing due to the workload implications.

*There were some practices who when they have negative results all the results are given out by reception (…). There were other practices that had a policy where HIV tests specifically were given out only by GPs, whether they were positive or negative, so then the thought of increasing the amount of HIV tests that they were going to have to manage was just insurmountable, so in a couple of those practices (…) I spent quite a long time talking about results management (…) giving them an opportunity to reflect on why they have that policy for an HIV test and not for other tests and whether that was really appropriate.*

Intervention deliverer

## Discussion

This qualitative study used NPT constructs to understand the social processes involved in acting on an educational intervention to increase HIV testing in primary care. For the first construct, coherence, or views and understanding of the intervention, we found that HCPs thought that the HIV educational intervention was necessary and relevant and experienced the delivery and content positively. Cognitive participation, the second construct, reflects the HCPs’ engagement with the intervention. Most HCPs felt more aware of the need to test for HIV, the importance of testing early and described greater knowledge of both HIV ICs and HIV prevalence of their practice population. Most HCPs felt more confident in their HIV knowledge, ability to talk to patients about HIV and to offer tests. Examples of collective action, the third construct, taken by HCPs following the training, include most HCPs being more likely to view HIV as a normal, routine test to offer based on indicator conditions. Importantly, changes were also described in relation to who and how tests were offered. When appraising the intervention, in line with the final NPT construct - reflexive monitoring - participants suggested that role-play exercises, observing consultations, more case examples and guidance on dealing with positive test results would be valuable and recommended follow-up sessions. An evaluation of changes in HIV testing rates found a small increase over the study period, but this could not be attributed to the educational intervention [30]. Our qualitative findings help interpret these quantitative results and demonstrate positive impacts of the intervention such as addressing testing capability and fulfilling the NPT constructs: coherence and cognitive participation. Interview data categorised under reflexive monitoring also highlighted several continued barriers to testing suggesting that more work to support improved motivation and opportunity for HIV testing is needed to drive collective action and increased testing rates.

### Comparisons with existing literature

The 3Cs and HIV study aimed to improve general practice staff skills and confidence to increase chlamydia and HIV testing rates [34], but had no impact on testing rates. The authors concluded that barriers preventing the translation of intentions into behaviour change remain [34]. In line with our findings, participants in the 3Cs and HIV study felt they needed additional training to develop and apply relevant communication strategies [35]. Others [36] have also found that HCPs struggle to remember to offer an HIV test, therefore interventions are needed which do not rely on HCPs remembering to test and increase testing opportunities (e.g. testing all new registrants). While in the current study, some HCPs perceived their testing focused less on patient risk factors and more on the presence of HIV ICs, this did rely on HCP’s memory.

In contrast, SHIP (Sexual Health in Practice), a multi-component educational intervention, achieved significantly increased HIV testing rates in general practices in a high-HIV prevalence area in London [25, 28]. Unlike the current intervention, SHIP involved two afternoons of training for GPs (and three for nurses) [25]. Chadwick et al. [36] also demonstrated significantly increased rates of HIV testing and a more universal approach to screening following the introduction of an electronic clinical decision support system.

Evidence suggests that more effective use of HIV ICs has the potential to trigger earlier HIV testing [37]. In support of this finding, a retrospective case-notes review in NHS City and Hackney Primary Care Trust of patients known to be HIV positive found that despite 51 out of 89 (57.3%) patients presenting to their GP with at least one HIV IC in the 2 years prior to diagnosis, only 17 (33.3%) were subsequently diagnosed with HIV by their GP [11]. Wellesley and colleagues (2015) emphasise the importance of education and training for primary care HCPs focused on common HIV IC seen in this setting [11]. This is supported by HCP accounts in the current study suggesting they lowered their threshold for offering tests based on increased awareness of HIV ICs. HCPs also reported that targeting testing towards those with indicator conditions reduced their concerns about offending patients. Furthermore, NICE guidelines on testing for HIV among all new registrants in high-HIV prevalence areas could mitigate against such concerns [26]. Pilots offering HIV tests to all new registrants or all patients attending the practice are feasible and acceptable [38]. A cluster-RCT promoting opt-out rapid HIV testing as part of registration health checks for new patients resulted in increased rates of HIV diagnosis [39]. This approach is also predicted to be cost-effective in the UK in the medium term [40]. Strong partnerships between specialist and primary care services are recommended to achieve high quality care and efficient referral processes for patients newly diagnosed with HIV [38]. Our findings highlighted the value of training providing useful information regarding specialist services. In this study, mixed views among HCPs were identified about pre-test counselling and obtaining informed consent prior to HIV tests. This issue has been debated [26, 41] and while some advocate for receiving informed consent [26], others argue that the process for offering HIV testing should not differ from any other test with similar implications [26]. Nurses and HCAs feeling that HIV testing is not part of their role has also been highlighted elsewhere [42].

### Strengths and limitations

This is the first time the online MEDFASH TIPs educational tool has been adapted for use as a face-to-face educational intervention. We achieved theoretical saturation from a diverse sample in relation to GP practice, professional role, gender and, years practising. All clinical staff in each practice were invited to receive the intervention which is a strength given the reported need for training among HCAs [35]. Conducting interviews at least 3 months after the intervention allowed examination of the impact on clinical practice and whether testing had been normalised into routine practice. However, some participants struggled to recall the intervention content. The use of NPT allowed for examination of issues with both the design of the intervention and its implementation. Understanding issues such as HCPs’ cognitive response and engagement with the intervention and practical barriers and facilitators to implementation will help others planning similar primary care-based interventions elsewhere.

## Conclusions

One-off training sessions on HIV testing, although experienced positively, are likely to require repetition and support from additional measures to help encourage increased HIV testing rates. Computer prompts based on risk algorithms are one additional strategy to support HIV testing. Prompts would notify the HCP when patients show HIV ICs or behavioural risk factors. Studies using clinical reminders or computer prompts based on HIV risk factors have shown the benefits of this type of intervention, impacting significantly on HIV testing rates [36, 43–48]. Increasing the uptake of HIV testing across healthcare settings [49] and reducing the stigma surrounding testing is still essential in the UK [2, 50]. Future interventions should therefore consider using behaviour change theory to develop complex interventions that address not only clinician’s capability to offer an HIV test, but also address issues of opportunity and motivation to enhance their effectiveness in increasing HIV testing [51, 52].

## Abbreviations

GUM: Genitourinary medicine
HCA: Healthcare assistant
HCP: Healthcare professionals
HIV ICs: HIV indicator conditions
HIV: Human immunodeficiency virus
MEDFASH: Medical Foundation for HIV and Sexual Health
NICE: National Institute for Health and Care Excellence
NPT: Normalisation Process Theory
TIPs: Testing In Practice
